# Systematic Analysis and Prediction of *In Situ* Cross Talk of O-GlcNAcylation and Phosphorylation

**DOI:** 10.1155/2015/279823

**Published:** 2015-10-27

**Authors:** Heming Yao, Ao Li, Minghui Wang

**Affiliations:** ^1^School of Life Science, University of Science and Technology of China, Hefei 230027, China; ^2^School of Information Science and Technology, University of Science and Technology of China, Hefei 230027, China; ^3^Centers for Biomedical Engineering, University of Science and Technology of China, Hefei 230027, China

## Abstract

Reversible posttranslational modification (PTM) plays a very important role in biological process by changing properties of proteins. As many proteins are multiply modified by PTMs, cross talk of PTMs is becoming an intriguing topic and draws much attention. Currently, lots of evidences suggest that the PTMs work together to accomplish a specific biological function. However, both the general principles and underlying mechanism of PTM crosstalk are elusive. In this study, by using large-scale datasets we performed evolutionary conservation analysis, gene ontology enrichment, motif extraction of proteins with cross talk of O-GlcNAcylation and phosphorylation cooccurring on the same residue. We found that proteins with *in situ* O-GlcNAc/Phos cross talk were significantly enriched in some specific gene ontology terms and no obvious evolutionary pressure was observed. Moreover, 3 functional motifs associated with O-GlcNAc/Phos sites were extracted. We further used sequence features and GO features to predict O-GlcNAc/Phos cross talk sites based on phosphorylated sites and O-GlcNAcylated sites separately by the use of SVM model. The AUC of classifier based on phosphorylated sites is 0.896 and the other classifier based on GlcNAcylated sites is 0.843. Both classifiers achieved a relatively better performance compared with other existing methods.

## 1. Introduction

Reversible posttranslational modification (PTM) can determine a protein's localization and activity state and interaction with other molecules by adding a modifying group or proteolytic cleavage [1]. Single PTMs are capable of changing the properties of proteins by adding new protein binding sites and abrogating protein-protein interaction [2]. However, many proteins are multiply modified and many evidences suggest that some of these modifications work together to accomplish a specific biological outcome [3, 4]. It is postulated that a code based on PTM may exist in proteins [5]. A typical example is the interplay between PTMs on histone polypeptides [5].

Cross talk between posttranslational modifications can be either positive or negative [2]. Positive cross talk means one PTM can act as a signal or be recognized by a binding protein that can add or remove the other PTM while negative cross talk happens when two different kinds of PTMs compete for the same residue (*in situ* cross talk) or one PTM masks the recognition site of the other PTM. Recent application of new mass spectrometric methods for O-GlcNAcylation and large-scale identification of phosphorylated sites show extensive and dynamic cross talk between O-GlcNAcylation and phosphorylation. Studies show that the level of O-GlcNAcylation within cells decreased after the treatment with phosphatase inhibitors and kinase activators [6, 7]. Further researches also demonstrate that O-GlcNAc/Phos cross talk functions in signaling, chronic diseases, transcription, and cell regulation [8–11]. Nevertheless, many questions regarding the general principles of the O-GlcNAc/Phos cross talk still remain unanswered. First, although there are many evidences to prove a reciprocal relationship of O-GlcNAc/Phos to signaling proteins and cytoskeleton proteins [8], some scholars still doubt the global existing of O-GlcNAc/Phos cross talk. For example, Trinidad et al. found that the cooccurrence of phosphorylation and O-GlcNAcylation is approximated to be random [12]. Second, as only part of the exact O-GlcNAc/Phos sites within a protein were identified and little systematic analysis was performed, the characteristics of O-GlcNAc/Phos cross talk sites and how they work are still unclear.

Today large amount of experimentally identified PTMs by mass spectrometry based proteomics experiment makes it possible to analyze PTM cross talk on larger scale. Many integrative studies have been conducted to analyze PTMs comparatively. For example, Peng et al. extracted several functional motifs with two PTM sites in close proximity [13] and Minguez et al. identified 35 pairs of different PTM types in total, suggesting a global network of functionally associated PTM types [14]. The study for predicting PTM cross talk sites is also emerging. YinOYang combined the predictions of O-GlcNAcylated sites and phosphorylated sites to discover potential O-GlcNAc/Phos cross talk sites [15]. Huang et al. built a naïve Bayes classifier integrating properties of PTM cross talk pairs predicting cross talks for pairwise combination of PTM sites [16].

In this study, we first conducted systematic analysis of O-GlcNAc/Phos cross talk* in situ* on serine (Ser) and threonine (Thr) using experimentally identified PTMs. We focused on evaluating their evolutionary conservation, collecting enriched GO terms of molecular functions and biological processes, extracting functional motifs, and analyzing interaction network and we found that* in situ* O-GlcNAc/Phos cross talk was specifically associated with several biological functions. After that, primary sequence and GO terms annotation were employed to predict O-GlcNAc/Phos cross talk sites. We proposed a feature selection algorithm that integrates minimal-redundant-maximal-relevant criterion with forward selection wrapper to deal with high-dimensional data. The code and data used in this study are freely available at: http://bioinformatics.ustc.edu.cn/glc-phos.

Finally, performance evaluation shows that our method achieved a relatively better performance and had the potential to help uncover the mystery of O-GlcNAc/Phos cross talk.

## 2. Materials and Methods

### 2.1. Data Collection

In this work, experimentally verified phosphorylated and O-GlcNAcylated sites on Ser and Thr were derived from several major databases, including Phospho.ELM [17] (version 9.0), PhosphoSitePlus [18] (version 2014_11), dbOGAP [19] (version 1.0), and dbPTM [20] (version 3.0). We extracted sites recorded having both O-GlcNAcylation and phosphorylation, regarding them as potential O-GlcNAc/Phos cross talk sites (O-GlcNAc/Phos sites). To avert interference that resulted from homology bias, the proteins primary sequences containing phosphorylated/O-GlcNAcylated sites were clustered by Blast and 70% threshold identity was used [21]. Then one protein from each cluster was selected randomly as a representative and remained for analysis.

The statistics for our final dataset were listed in Table 1. From the table, we can find that about half of O-GlcNAcylated proteins have O-GlcNAc/Phos sites. It is consistent with the fact that O-GlcNAcylation tends to occur at sites on the protein backbone that are similar to those modified by protein kinases, which supports the hypothesis that O-GlcNAcylation has a global reciprocal relationship with phosphorylation.

### 2.2. Gene Ontology Enrichment Analysis

With the use of PANTHER [22] for GO enrichment analysis, proteins containing O-GlcNAc/Phos sites were uploaded as foreground to find the most relevant GO terms and three sets of proteins were used as background separately (see details in Section 3). A two-tailed Fisher exact test with Bonferroni correction for multiple testing was conducted to calculate the *p* value of each annotation. Nonredundant GO terms in biological process, molecular function, and cellular component with enrichment ratios >4 and a *p* value < 0.05 were selected.

### 2.3. Evolutionary Analysis

To analyze the conservation of sites containing O-GlcNAc/Phos cross talk or sites modified by one of them alone, we download 89706, 52490, 29249, 25488, 24208, 41136, 17655, 20131, 26083, and 22693 proteins for* Homo* (human),* Mus* (mouse),* Canis lupus familiaris* (dog),* Rattus norvegicus* (rat),* Bos taurus* (bovine),* Danio rerio* (zebrafish),* Gallus* (chick),* Pan troglodytes* (chimpanzee),* Sus scrofa* (pig), and* Equus* (horse) from Uniprot database [23] (version: 2015_04). As previously reported, the approach mainly based on sequence similarity [24] was adopted by detecting orthologs among the ten species. Each group of orthologous protein sequences was multialigned by MUSCLE [25].

**Figure 1:**
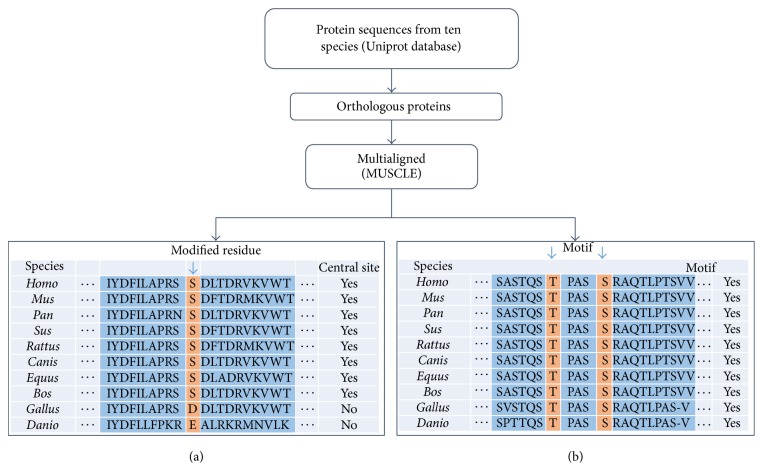
The workflow to calculate the evolutionary conservation score of modified sites and motifs. Protein sequences of ten species were downloaded from Uniprot database and then each group of orthologous proteins was multialigned by MUSCLE software. (a) Conservation scores of modified sites were calculated. (b) After extracting overrepresented motifs, proteins containing these motifs were selected and conservation of motifs in these proteins was calculated.

Generally, people assume that the conservation of an amino acid site can serve as a good approximation for the conservation of PTM on that site [14]. In this work, we used the Residue Conservation Ratio (RCR) as the conservation score of each site and it was calculated as below:

(1) $$\begin{array}{c} \text{RCR}=\frac{N_{c}}{N_{total}}, \end{array}$$

where *N*_*c*_ is the number of specific residues occurring on the site under study and *N*_total_ is the number of sequences we multialigned in each orthologous proteins group (shown in Figure 1(a)). As both the overall conservation of the protein and the location of these sites may have a significant impact on conservation scores, we calculated the RCRs for 41 residues around the site under study (±20 residues) to build the reference distribution of conservation for the flanking regions. Then the RCR of the modified site was mapped to calculate the percentile of its value, which was named as relative RCR (rRCR). The distributions of RCRs and rRCRs of sites containing cross talk were compared with that of sites having only one PTM by means of a nonparametric Kolmogorov-Smirnov test.

### 2.4. Motif Extraction and Network Analysis

In this study, we adopted the workflow first proposed by Peng et al. [13] to extract functional motif. We extracted sequences centered on the modified residues and extended them to 21 amino acids (±10 residues). Since sites near the N- or C-terminal cannot be extended, we used “*∗*” to replace them. We used motif-X software [26] to find out the overrepresented motifs and then selected all proteins in the dataset containing these motifs. These motifs were firstly filtered by conservation score of corresponding sites in each protein.

The method of calculating the conservation score of each motif is shown in Figure 1(b). Proteins with conservation scores <0.4 were filtered out. Proteins containing different overrepresented motifs were further analyzed for annotation enrichment using PANTHER and the background was the set of proteins containing O-GlcNAc/Phos sites. Only proteins significantly enriched in GO biological process or GO molecular function (*p* value < 0.05) were regarded as containing a potential functional motif and selected for further network analysis.

The network analysis was performed by the use of GeneMANIA [27]. A list of proteins under study was uploaded to produce respective gene interaction network (automatically selected weighting method). The network included coexpression, colocalization, physical interaction, and genetic interaction and 10 related genes were also displayed. False discovery rate (FDR) is provided to each function prediction which is greater than or equal to the possibility that this is a false positive.

### 2.5. Dataset for Site Prediction

Our dataset consisted of O-GlcNAc/Phos sites, only O-GlcNAcylated sites, and only phosphorylated sites in human. Although the conclusion about only O-GlcNAcylated or only phosphorylated sites cannot be made with certainty which may include true but not yet identified cross talk sites, we assumed that they were enough to generalize the characteristics of real only O-GlcNAcylated sites or only phosphorylated sites.

To train a classifier based on phosphorylation (phos-based classifier), we regarded the potential cross talk sites as positive samples and the only phosphorylated sites as negative samples. The ratio of negative/positive samples was 10 : 1 considering the large number of phosphorylated sites. Similarly, to train a classifier based on O-GlcNAcylation (O-GlcNAc-based classifier), we used the potential cross talk sites as positive samples and the only O-GlcNAcylated sites as negative samples. In this case, the ratio of negative/positive samples was 2 : 1, which was the rough ratio of only O-GlcNAcylated/phos potential cross talk sites in our collection.

### 2.6. Feature Extraction and Coding

The primary sequence is always an important feature for site prediction. We extracted 21 residues (±10 residues) around the modified site as its sequence context and then these 21 residues were converted into a vector of binary values using the sparse encoding method [19]. In this method, each type of residues was coded with 21 binary values, for example, 100⋯0 (one followed by 20 zeros) for Cys and 010⋯0 for Ser. Thus, for each modified site under study, the dimension of feature vector representing its sequence context was 21*∗*21.

Considering that proteins with cross talk may be significantly enriched in several specific functions, we downloaded GO annotation file for human proteins and extracted GO terms associated as features. Finally, the functional information was expressed as a 15404-dimensional vector of binary values. Each dimension represented one GO term where the value “1” meant the protein containing this site annotated with corresponding GO term.

### 2.7. Feature Selection and Site Prediction

We proposed a two-stage feature selection algorithm that incorporated minimal-redundant-maximal-relevant criterion [28] and forward selection wrapper to obtain the optimal feature subset. Given two random variables *x* and *y*, their mutual information is defined as

(2) $$\begin{array}{c} I\left(\;x;y\;\right)=\overset{}{\underset{}{\iint }}p\left(\;x,y\;\right)\log\frac{p\left(x,y\right)}{p\left(x\right)p\left(y\right)}dx\;dy, \end{array}$$

where *p*(*x*),** *p*(*y*), and *p*(*x*, *y*) are their probabilistic density functions.

The function *R* was used to evaluate the redundancy between two selected features and function *D* reflects the relevance between features and classifications. Max-relevant is to search features satisfy (2) and min-redundant features satisfy (3). Consider

(3) $$\begin{array}{c} \max D\left(\;S,c\;\right),\;D=\frac{1}{\left|S\right|}\overset{}{\underset{x_{i}\in S}{\sum }}I\left(x_{i};c\right), \end{array}$$

(4) $$\begin{array}{c} \min R\left(\;S\;\right),\;R=\frac{1}{{\left|S\right|}^{2}}\overset{}{\underset{x_{i},x_{j}\in S}{\sum }}I\left(x_{i},x_{j}\right). \end{array}$$

The “minimal-redundant-maximal-relevant” criterion can be expressed as the following simplest form:

(5) $$\begin{array}{c} \max Ф\left(\;D,R\;\right),\;Ф=D-R. \end{array}$$

At first, MRMR was proposed to rank GO features and obtain a subset that consisted of top 500 ranked GO features, which can be used for wrapper forward feature selection later. Secondly, we used primary sequence features to train the first SVM model (implemented by LIBSVM package), mainly serving as a baseline. Then we started to add GO features one by one from higher to lower rank score to train our model and, at the meantime, AUCs were calculated and recorded for performance evaluation. The procedure was continued until all GO features selected had been added. From the recorded AUCs, the feature subset corresponding to the maximal AUC was used to train our final SVM model. We chose the radial kernel and adopted the grid search strategy to obtain optimized parameters—cost and gamma. Considering the unbalance of our dataset, the weight parameter of negative samples was set as negative/positive samples ratio. By the use of SVM model, a possibility will be returned when predicting a site. The higher the possibility, the more likely this site containing O-GlcNAc/Phos cross talk.

### 2.8. Performance Assessments

To evaluate the classifier performance, receiver operating characteristic (ROC) curve and the area under ROC curve (AUC) were adopted with 10-fold cross-validation. Accuracy (Acc), sensitivity (Sn), specificity (Sp) and Matthew's Correlation Coefficient (MCC) were also used to quantify the performance and they were defined as follows:

(6) Acc=TN+TPTN+TP+FN+FP,Sn=TPTP+FN,Sp=TNTN+FP,Pre=TPTP+FP,MCC=TP×TN−FP×FNTP+FN×TP+FP×TN+FN×TN+FP,

where TN, TP, FN, and FP represent true negative, true positive, false negative, and false positive, respectively.

## 3. Results

### 3.1. GO Enrichment Analysis

**Figure 2:**
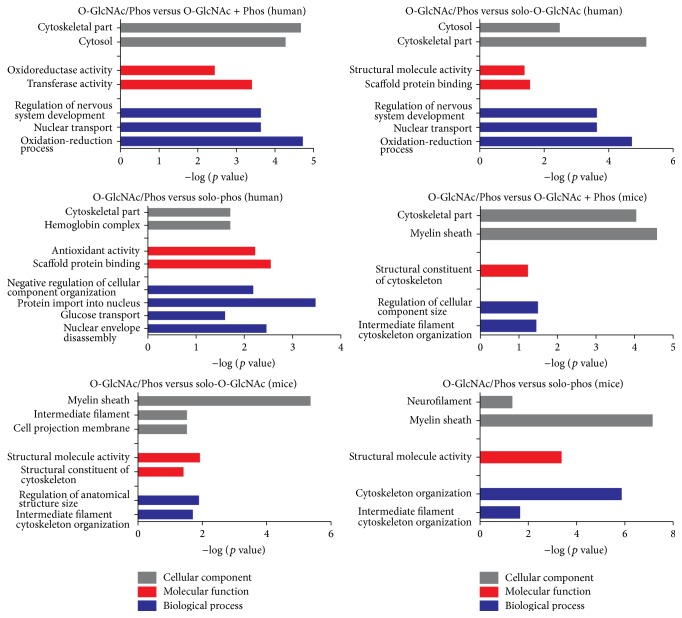
Gene ontology annotation enrichment analysis. The enriched biological process, molecular function, and cellular component GO terms for proteins containing O-GlcNAc/Phos cross talk.

First, to obtain GO terms specific to the cross talk we used human proteins having* in situ* O-GlcNAc/Phos sites as foreground and human proteins both phosphorylated and O-GlcNAcylated but not in the same site (O-GlcNAc + Phos) as background. The result is shown in Figure 2. Clearly, proteins with O-GlcNAc/Phos sites were significantly enriched in oxidation-reduction process (*p* value: 1.89*E* − 05), nuclear transport (*p* value: 2.32*E* − 04), and regulation of nervous system development (*p* value: 2.32*E* − 04). Due to the limited number of proteins with O-GlcNAc + Phos, we also used proteins only having O-GlcNAcylation and phosphorylation as background separately to avoid any possible bias in dataset. The results show that, compared with only O-GlcNAcylated or phosphorylated proteins, proteins having O-GlcNAc/Phos sites were also associated with nuclear transport, cytoskeleton, structure molecular activity and oxidation-reduction process. The analysis of mice proteins showed the same thing. Mice proteins having O-GlcNAc/Phos were significantly enriched in cytoskeleton organization when compared with three lists of background proteins described above. These results imply that O-GlcNAc/Phos sites play roles in several specific functions, such as cytoskeleton organization and nuclear transport, where the interaction between O-GlcNAcylation and phosphorylation may matter.

### 3.2. Evolutionary Analysis

**Figure 3:**
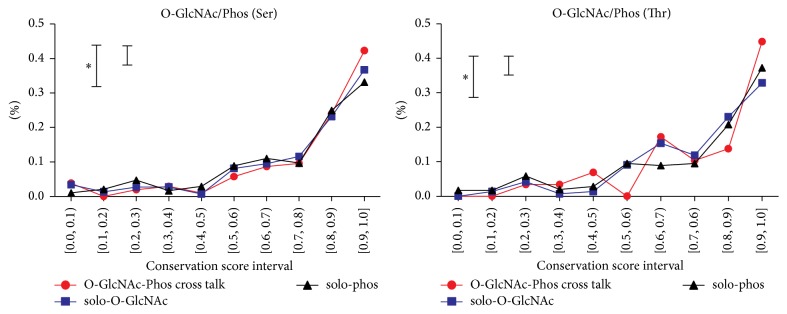
Conservation evaluation. The distribution of conservation score of proteins with O-GlcNAc/Phos sites, only O-GlcNAcylated sites, and only phosphorylated sites in human and in mice.

Figure 3 shows the distribution of conservation score of proteins with O-GlcNAc/Phos sites, only O-GlcNAcylated sites, and only phosphorylated sites in human. By using Kolmogorov-Smirnov test, we found there was no difference between conservation of O-GlcNAc/Phos sites and only GlcNAcylated sites although O-GlcNAc/Phos sites were slightly more conserved than only phosphorylated sites (*p* value: 9.8*E* − 02). Moreover, further comparison of rRCR distribution and the study of proteins in mice also showed that O-GlcNAc/Phos sites were not under obvious additional evolutionary pressure, which were consistent with previous studies. Trinidad et al. suggested that there is little evolutionary pressure for the* in situ* O-GlcNAc/Phos sites based on their observation that the cooccurrence of phosphorylation and O-GlcNAcylation is approximated to be random [12]. Pan et al. performed bioinformatics analysis of the* in situ* cross talk of Tyr modification and they also concluded that no additional natural selection on multiply modified residues [29].

### 3.3. Motif Extraction and Network Analysis

**Figure 4:**
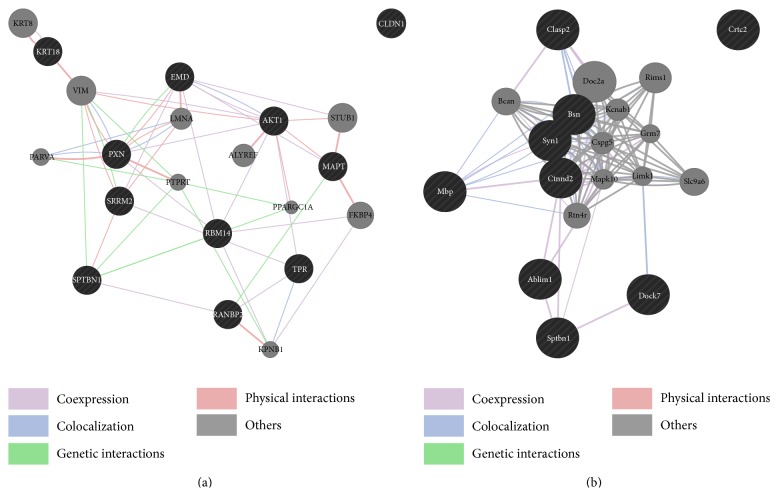
Two examples of protein network for extracted motifs. A list of gene names corresponding to proteins containing overrepresented motifs was uploaded to GeneMANIA (black nodes). 10 related genes were predicted (gray nodes). (a) Motif: PxxS. (b) Motif: TxxxS.

After using motif-X and filtering our result, we finally discovered three potential functional motifs (details in Table 2 and Table S1 in Supplementary Material available online at http://dx.doi.org/10.1155/2015/279823). We examined the biological process and molecule function categories associated with motifs and find Pxx[S] significantly enriched in endomembrane system organization and GTPase binding, Txxx[S] enriched in cytoskeleton organization, and [T]xxxxxxxxxP to take part in regulation of cell cycle. After that, we evaluated whether proteins containing the same motif are part of a single protein network. As shown in Figure 4, for Pxx[S] and Txxx[S] we obtained well-defined networks and the functions predicted by GeneMANIA were consistent with the enriched GO terms of each motif described above.

To validate the discovered motifs, we further searched published literature. We found that many proteins involved in cell motility and cytoskeleton were reported to contain Txxx[S] motif, as well as some scaffold proteins and GTPase. TxxxS is the GSK3 consensus phosphorylation motif and the phosphorylation by GSK3 can result in proteolytic cleavage or a full degradation. GSK3 does not recognize its targets on the basis of simple primary sequence recognition but a priming phosphorylation event is required [30]. In Txxx[S] motif, phosphorylation of the Ser by another kinase acts as the priming event and leads to the fact that GSK3 phosphorylates the threonine [31]. These facts elucidate roles of O-GlcNAc/Phos competition and switch on Ser in protein proteolytic processing. The occupancy of O-GlcNAc on Ser inhibits the following phosphorylation on Thr, and thus protein proteolysis is avoided. Correspondingly, the phosphorylation of the Ser can lead to protein proteolysis.

Ser/Thr near Pro are also reported to have the ability to modulate the structural deformations caused by the pyrrolidine ring of Pro, which can allow or prevent specific interactions to take place [32]. This fact corresponds with our finding that Pxx[S] may have played a role in regulation of protein complex disassembly. In summary, the extracted motifs we assumed functional using GO enrichment can be supported by previous study.

### 3.4. Evaluation of GO Features

After bioinformatics analysis, we found that proteins having O-GlcNAc/Phos cross talk were enriched in some specific GO terms, which implies that the use of functional information may efficiently improve the performance of O-GlcNAc/Phos cross talk sites prediction. Due to the huge number of GO terms, a reasonable feature selection strategy is critical for the prediction performance of our method.

**Figure 5:**
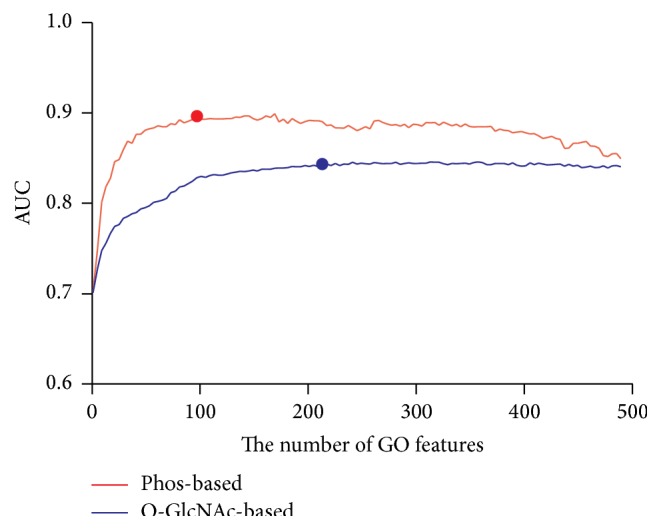
Comparison of ROC curve with different number of GO features. *x*-axis represents the number of ranked GO features added to sequence features, and *y*-axis represents the AUC of the corresponding models.

To evaluate GO information, we selected top 500 GO terms ranked by MRMR and added them iteratively from top 1 to top 500. During the feature selection process, the AUC results of different number of GO features were calculated and shown in Figure 5. It clearly shows that the prediction performance was significantly improved after the addition of a small number of GO features and then it grew slowly until it arrived at the peak. For phos-based classifier, the AUC is 0.702 when only sequence features were used and the AUC boosted to 0.801 when 10 GO feature were added. After the addition of 40 GO features, the AUC arrived at 0.877 and then the increase of AUC with more GO features was not dramatic as before. Finally, the optimal AUC reached 0.896 when using 98 GO features. After that, the AUC experienced a slight decrease with the addition of more GO terms. For phos-based classifier, the AUC started with 0.701 and increased to 0.773 when using 20 GO features and 0.832 when using 100 GO features. Finally, the optimal AUC was 0.843 after adding 218 GO features and then the AUC kept constant with more GO features though small fluctuations existed. Top 10 GO terms associated with phos-based classifier ranked by MRMR and top 20 GO associated with O-GlcNAc-based classifier were listed in Table S2. Intriguingly, nearly half of these GO terms, for example, scaffold protein binding, were enriched in O-GlcNAc/Phos cross talk proteins, which corresponded to our results in GO enrichment analysis.

**Figure 6:**
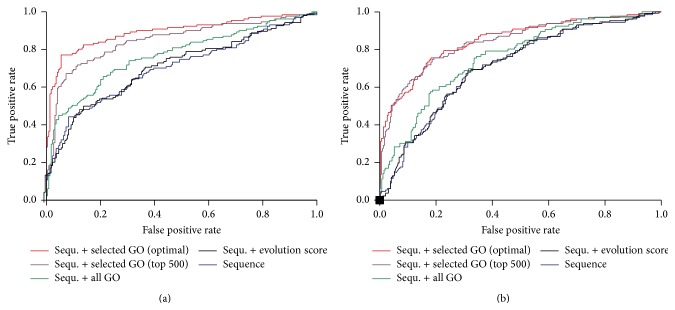
ROC curves of 10-fold cross-validation performance using different feature subsets. Sequence/Sequ.: primary sequence context; all GO: all GO terms; selected GO (optimal): the optimal subset of GO features obtained in Figure 5; selected GO (top 500): top 500 GO terms ranked by MRMR. (a) Phos-based classifier. (b) O-GlcNAc-based classifier.

For further investigations of our feature selection method, we compared the ROC curves of 10-fold cross-validation performance with different groups of features (Figure 6). The performances of SVM with only primary sequence features were usually poor and it is consistent with the previous finding that O-GlcNAc/Phos sites do not display a definite consensus sequence generally. The ROC curve after adding conservation scores stayed the same and it further substantiated our observation that O-GlcNAc/Phos sites show no signature of additional evolutionary pressure. While the use of all GO features can make the situation slightly better, the use of selected GO terms by MRMR and wrapper can significantly improve the performance. These results verified the efficiency of our feature selection method.

### 3.5. Performance Evaluation

Using optimal feature subsets, the AUC of phos-based classifier is 0.896 while the AUC of O-GlcNAc-based classifier is 0.843. In addition to AUC, we also employed other performance measurements to evaluate the classifiers, such as Acc, Sn, Sp, Pre, and MCC. The performance was evaluated at high, medium, and low stringency levels, which correspond to Sp > 0.95, 0.90, and 0.85, respectively, and the results are shown in Table 3. From the results, we can find that the contribution of sequence features and GO features is consistent and remarkable. For example, at medium level, the Sn of phos-based classifier is 0.778 and the MCC is 0.668. It should be noticed that the number of false positive instances is somewhat rough because of the uncertainty of the non-O-GlcNAc/Phos sites which have been explained before.

To further evaluate the prediction performance of phos-based classifier and O-GlcNAc-based classifier, we compared it with the other existing O-GlcNAc/Phos cross talk site prediction tool: YinOYang [15]. The YinOYang program is based on a neural network trained on 40 experimental determined O-GlcNAcylated sites. It can predict potential O-GlcNAc/Phos sites when linked to the NetPhos, which can recognize phosphorylated sites. The testing datasets were the same as that we used to evaluate our method and the same evaluation procedure mentioned above was adopted. As the information whether the site can be phosphorylated or O-GlcNAcylated was used in our method to predict a O-GlcNAc/Phos cross talk site, one testing dataset was a combination of sites containing O-GlcNAc/Phos cross talk and sites having only phosphorylation, while the other testing dataset was a combination of cross talk sites and only O-GlcNAcylated sites. The results in Table 3 show that the Sns and MCCs at high, medium, and low levels of both phos-based classifier and O-GlcNAc classifier are better than these of YinOYang which implies our method is efficient.

### 3.6. Predicted O-GlcNAc/Phos Cross Talk Sites

Using the classifiers we constructed, we predicted our dataset by 10-fold cross-validation. The entire results are freely available at http://bioinformatics.ustc.edu.cn/glc-phos. The threshold was chosen under the highest AUC and all sites were ranked by the possibilities. As we have discussed, the conclusion about only O-GlcNAcylated or only phosphorylated sites in our dataset cannot be made with certainty which may include true but not yet identified cross talk sites. Thus, we further analyzed the top 10 potential* in situ* O-GlcNAc/Phos cross talk sites provided in Table 4 that were annotated with only O-GlcNAcylation. Interestingly, we found 7 out of 10 sites containing a phosphorylation in the adjacent site (±5 residues) while from our dataset only about 1/3 O-GlcNAcylated sites had an adjacent phosphorylation. We also analyzed the top 10 potential* in situ* O-GlcNAc/Phos cross talk sites that were annotated with only phosphorylation in Table S3. Although only 1 site was found containing an adjacent O-GlcNAcylation, it made sense considering only a small number of O-GlcNAcylated sites were identified while a large number of phosphorylated sites were detected.

The result suggests that the O-GlcNAc/Phos cross talk in adjacent site may have similar characteristics to the O-GlcNAc/Phos cross talk at the same site as the construction of our classifiers depends mainly on the primary sequence and GO terms. We did not perform the study of O-GlcNAc/Phos cross talk in adjacent sites because of the limitation of our dataset; thus further analysis is needed to prove our assumptions and our study will provide useful guidance.

## 4. Discussion

It is reported that O-GlcNAc/Phos cross talk is a very complex issue that contains the interplay between O-GlcNAcylation and phosphorylation on the same site, adjacent sites, and even on different proteins [8]. In this study, we only focused on* in situ* O-GlcNAc/Phos cross talk considering the quality of current dataset. Following previous study [13, 29], we roughly assumed a site with both O-GlcNAcylation and phosphorylation as a potential* in situ* O-GlcNAc/Phos site as the competitive site occupancy will happen when they coexist. However, more interference will be introduced if we make a similar assumption to potential O-GlcNAc/Phos cross talk in adjacent sites. The interplay may not occur even when they cooccur because of various factors such as space conformation. As convincing and reliable results depend on the dataset of good quality, we only performed the systematics analysis on* in situ* O-GlcNAc/Phos sites. Our work has just begun to elucidate unanswered questions described in Section 1 from another angle.

The selection of database ensures the completeness and reliability of our dataset. The Phospho.ELM, PhosphoSitePlus, and dbPTM are the most commonly used and major databases in the world. In addition, we also extracted data in dbOGAP, which is designed specifically for collecting O-GlcNAcylated proteins and sites. These databases all integrate both low- and high-throughput (LTP and HTP) data source [17–20].

From the GO enrichment analysis, we obtained the functions specific to the fact of* in situ* O-GlcNAc/Phos sites; our results showed that proteins having* in situ* O-GlcNAc/Phos sites were significantly enriched in nuclear transport, cytoskeleton, and structure molecular activity. It not only substantiates the overall functional role of O-GlcNAc/Phos cross talk but also enlightened us that the GO terms hold the potentiality to improve the performance in site prediction, which drove our investigation on site prediction method in the second part and made a great contribution to prediction performance.

Our evolution analysis showed no additional evolution pressure on sites having O-GlcNAc/Phos cross talk, implying that the conservation of cross talk sites may depend more on PTM types. Combined with the result in GO enrichment analysis, we can conclude that the functional role of O-GlcNAc/Phos site may not directly lead to an increase in site conservation. It makes sense as previous study shows that the O-GlcNAc/Phos cross talk tends to play roles in chronic diseases rather than fatal diseases.

Then, we extracted 3 functional motifs. The workflow we used reduced the possibility of false discoveries and literature finding also proved the reasonability of the outcomes. Our following investigation of these motifs gave us more understanding of the underlying mechanism. Although it should be noted that the statistical significance cannot guarantee a biological importance, our results provide sensible hypotheses for follow-up experiments, which may lead to a further confirmation of our predictions. On the other hand, as only 3 motifs were extracted, it is suggested that* in situ* O-GlcNAc/Phos sites may not follow a stringent recognition motif, which also suggests that a traditional site prediction strategy using primary sequence may have a poor predictive outcome.

Site prediction is an intriguing and significant topic in the study of PTMs because it is a prospective way to overcome the high-cost and labor-intensive shortcomings of PTM site identification using experimental techniques. However, the development of computational approaches in identifying O-GlcNAc/Phos sites is still immature. Current approaches only take the advantages of primary sequences and the predictive performances are usually not ideal [15, 33].

Our bioinformatics analysis gave us important clues to the feature selection and led to satisfying predictive performance. In this work, we integrated primary sequence features and GO features by using MRMR criterion and then adopted statistics learning method to predict O-GlcNAc/Phos cross talk sites. The following performance evaluation showed that our method achieved relatively high sensitivity comparing with other existing tools. Due to the development of mass spectrometry, a large amount of phosphorylated sites was experimentally verified. In addition, the computational prediction of phosphorylation sites and O-GlcNAcylated sites is continuously improving [33–36]. Thus, combined with experimentally verified or computationally predicted singly modified sites, our phos-based classifier and O-GlcNAcylated-based classifier can be used for proteomic-wide screening and systematic examination for* in situ* O-GlcNAc/Phos cross talk sites.

## 5. Conclusion

In this work, by using bioinformatics tools, we found that proteins having O-GlcNAc/Phos were significantly enriched in nuclear transport, cytoskeleton, and structure molecular activity. Although no additional conservation was found in O-GlcNAc/Phos sites, 3 functional motifs were extracted. For one thing, these findings give us more understanding on general characteristics and potential mechanism of O-GlcNAc/Phos cross talk and also provide sensible hypotheses for follow-up wet experiments. For another thing, the results drove our further research on site prediction. The integration of GO terms with primary sequence features and the introduction of MRMR criterion into feature selection workflow lead to a significant promotion in predictive performance of O-GlcNAc/Phos cross talk sites. It is undoubtedly a progress in the development of sensitive computational tools to discover O-GlcNAc/Phos cross talk sites. Thus, it is anticipated that the classifiers constructed in this work could be useful for biomedical research and guide the related experimental validations.

In this study we only focused on* in situ* O-GlcNAc/Phos cross talk on Ser and Thr. Currently, lots of publications also demonstrate the common collaboration of PTMs near each other [13, 37, 38] as well as the interplay of PTMs on different proteins, for example, the O-GlcNAcylation of phosphate cycling enzymes. It is frequent that PTMs occur in close proximity [39, 40], but the interplay among them is more elusive since more factors may be involved. Our analysis in predicted site revealed that O-GlcNAc/Phos cross talk in adjacent sites might share something in common with* in situ* O-GlcNAc/Phos cross talk. In addition, many publications show that cross talk of O-GlcNAcylation and phosphorylation in close proximity play roles in several signaling pathways [11, 41–43]. Thus, further investigation of cross talk of O-GlcNAcylation and phosphorylation in close proximity becomes more compelling and it may be potential to uncover the mechanism of signaling regulation by GlcNAc/Phos cross talk.

## Supplementary Material

Table S1: It lists the extracted motifs from motif analysis and relevant information of sites containing the motif. Enriched GO terms obtained from GO enrichment analysis and GeneMANIA analysis are also contained.
Table S2: Top 10 GO terms associated with phos-based classier ranked by MRMR and top 20 GO associated with O-GlcNAc-based classier are listed.
Table S3: Top 10 potential in situ O-GlcNAc/Phos cross talk sites that were annotated with only phosphorylation are listed.

## Figures and Tables

**Table 1:** The statistics for data we collected from major PTM database.

Species	Modification	Residues	Number of sites	Number of proteins
Human	Phosphorylation	S	115792	13575
		T	51802	13575
	O-GlcNAcylation	S	250	118
		T	173	84
	O-GlcNAc/Phos	S	103	55
		T	29	23

Mice	Phosphorylation	S	55993	10309
		T	16978	6764
	O-GlcNAcylation	S	484	248
		T	386	203
	O-GlcNAc/Phos	S	116	76
		T	30	27

**Table 2:** Summary of motifs extracted from peptides containing PTM cross talk and their enriched function.

Cross talk in motif	Motif	GO biological process	GO molecular function	GeneMANIA function prediction
O-GlcNAc-Phos	Pxx[S]	Negative regulation of protein complex disassembly (2.8*E* − 4)	Cytoskeletal protein binding (9.9*E* − 3)	Tubulin binding (3.3*E* − 02); microtubule cytoskeleton organization (3.3*E* − 02)

O-GlcNAc-Phos	Txxx[S]	∖	Cytoskeletal protein binding (9.7*E* − 3)	Axon part (2.2*E* − 03); cognition (4.6*E* − 02)

O-GlcNAc-Phos	[T]xxxxxxxxxP	Positive regulation of cell cycle (5.1*E* − 02)	∖	Response to peptide hormone (2.2*E* − 09)

**Table 3:** Performance evaluation.

Classifier	Level	Sp (%)	Sn (%)	ACC (%)	Pre (%)	MCC (%)
Phos-based	High	94.8	70.0	88.3	83.8	69.0
	Medium	90.0	77.8	86.7	74.2	66.8
	Low	85.0	82.0	84.2	66.9	63.2

YinOYang *∗*1	∖	75.5	42.7	68.9	67.5	32.7

O-GlcNAc-based	High	94.9	50.4	83.0	78.8	63.3
	Medium	90.0	57.3	81.2	67.9	50.0
	Low	85.2	70.2	81.0	63.3	53.5

YinOYang *∗*2	∖	71.2	42.7	56.8	59.6	14.8

For YinOYang *∗*1 and YinOYang *∗*2, we used the same method to evaluate the performance of YinOYang while the test sets were different. For YinOYang *∗*1, the test dataset is the same with that used for phos-based classifier and, for YinOYang *∗*2, the test dataset is the same with that used for O-GlcNAc-based classifier.

**Table 4:** Information of top 10 potential *in situ* O-GlcNAc/Phos cross talk sites using O-GlcNAc-based classifier.

Protein	Site	Gene	Residue	Probability	Note
P68871	85	HBB	T	0.61	Y
P49790	1179	NUP153	T	0.61	Y
Q6PJT7	370	ZC3H14	T	0.60	N
P51610	806	HCFC1	S	0.59	Y
P51610	726	HCFC1	T	0.58	Y
Q96KR1	148	ZFR	S	0.58	Y
O00151	94	PDLIM1	T	0.55	N
Q86X29	316	LSR	T	0.54	Y
Q3MIT2	171	PUS10	S	0.54	Y
Q9NYV4	592	CDK12	T	0.53	N

Note: “Y” means an adjacent phosphorylation is found while “N” means not.
